# Complete mitochondrial DNA analysis of *Hydrotaea ignava* (Insecta, Diptera, Muscidae)

**DOI:** 10.1080/23802359.2017.1407703

**Published:** 2017-11-24

**Authors:** Mustafa Zafer Karagozlu, Seong Hwan Park, Sang-Eon Shin, Chang-Bae Kim

**Affiliations:** a Department of Biotechnology, Sangmyung University, Seoul, Korea;; b Department of Legal Medicine, Korea University College of Medicine, Seoul, Korea

**Keywords:** Insecta, diptera, muscidae, complete mitochondrial genome, *Hydrotaea ignava*

## Abstract

The complete mitochondrial genome sequenced and analyzed from a black garbage fly, *Hydrotaea ignava* which is critically important species for forensic investigations. The size of mitochondrial genome is 17,026 bp with 40.8% A, 11.2% C, 8.2% G and 39.8% T distribution. This is the longest within complete mitochondrial genome records of the Muscidae species. The mitochondrial genome is composed of 13 protein coding, two ribosomal RNA and 22 tRNA genes and a putative control region. Furthermore, phylogenetic relationships of the superfamily Muscoidea evaluated due to mitochondrial protein coding genes. The results showed that the *H. ignava* placed in the paraphyletic Muscidae family and early diverged from a clade including Muscidae, Anthomyiidae and Scathophagidae species. This is the first complete mitochondrial genome for the genus *Hydrotaea*.

*Hyrotaea ignava* is a Muscidae species which formerly known as *Ophyra ignava*. Mostly they appear at ammoniacal fermentation stage (Couri et al. 2009) and their larvae are predators for the other dipteran larvae in the third stage of decay (Byrd and Castner 2001). Therefore, identification of the species from corpse is critically important for forensic investigations but it is hard to identify them by morphology. Especially, larvae of *H. ignava* are very similar with the of *H. aenescens* (Ishijima 1967). One of the most useful methods to species identification is DNA barcoding method. However, barcoding can fail to separate members of closely related species groups and morphologically similar species (Hajibabaei et al. 2007). In this context, sequencing of complete mitochondrial genome can overcome these difficulties. Nevertheless there is no complete mitogenome record for the genus *Hydrotaea*. In this study, we are providing the complete mitochondrial genome of *H. ignava* which is the first complete mitochondrial genome belonging to the genus.

The adult *H. ignava* specimen were collected from shade, forest, Dukso-eup, Namyangju-si/Korea 37°58′12.5″N, 127°24′14.9″ E, August 2013. The specimens were identified by DNA barcoding (Park and Shin 2013) and deposited in Department of Legal Medicine, Korea University (16Ig02). Whole genomic DNA was extracted from the legs and thorax. Methods for sequencing complete mitogenome and analysis of phylogenetic tree were described previously (Karagozlu et al. 2016).

The length of complete mitogenome of *H. ignava* (KY977435) is 17,026 bp. The structure and orientation is identical with ancestral insect genome (Cameron 2014). It has 335 bp of intergenic nucleotides, in 18 locations, with intergenic spacer lengths from 2 to 78 bp. There are 15 overleaping regions, with overlap lengths ranging from 1 to 62 bp. The putative control region of *H. ignava* (2,160 bp) is also located between 12S rRNA and tRNA^Ile^. Total six of 13 protein coding genes use ATG as starting codon. They are *cox2*, *atp6*, *cox3*, *nad4*, *nad4l* and *cytb*. Besides, *atp8*, *nad3*, *nad5*, *nad6* and *nad2* use ATT for starting codon. Only *nad1* and *cox1* use different starting codons that are ATA and TCG, respectively. A total 10 out of 13 genes stop with TAA codon. Exceptionally, *nad1* and *cytb* use TAG as stop codon. There is only *cox2* gene uses incomplete stop codon.

The genus *Ophyra* was synonymized *Hydrotaea* (Huckett and Vockeroth 1987). The position of *Ophyra* has been subjected for a long time and still uncertain (Patitucci et al. 2010). The recent molecular-based phylogenetic analysis suggests that *Ophyra* and *Hydrotaea* are located in the subfamily Muscinae individually (Schuelli et al. 2007). Hence, phylogenetic relationship of *H. ignava* in the superfamily Muscoidea was investigated (Figure 1). The results showed that the *H. ignava* placed in the paraphyletic Muscidae family and early diverged from a clade including Muscidae, Anthomyiidae and Scathophagidae species. Unfortunately, there is no complete mitogenome record from *Ophyra* and *Hydrotaea* to confirm their relationship additional complete mitogenome data from both genera should provide. The present study presents additional data for molecular Muscoidea phylogeny.

**Figure 1.**
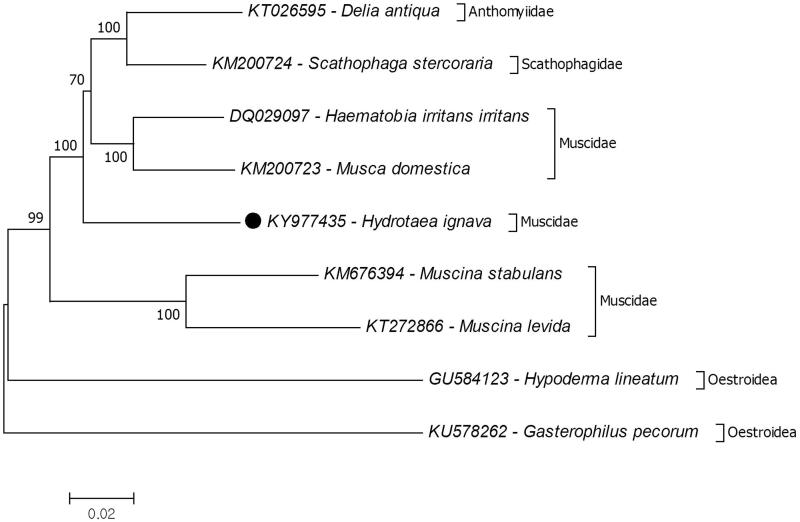
Phyloegenetic relationship of *Hydrotaea ignava* in the superfamily Muscoidea. The two Oestidae species records belonging to the superfamily Oestroidea selected as representative of the outgroup. The complete mitochondrial genomes were retrieved from the GenBank for reconstruction of phylogenetic tree.
